# Multilocus Sequence Typing and Virulence-Associated Gene Profile Analysis of *Staphylococcus aureus* Isolates From Retail Ready-to-Eat Food in China

**DOI:** 10.3389/fmicb.2018.00197

**Published:** 2018-03-13

**Authors:** Xiaojuan Yang, Shubo Yu, Qingping Wu, Jumei Zhang, Shi Wu, Dongli Rong

**Affiliations:** State Key Laboratory of Applied Microbiology Southern China, Guangdong Provincial Key Laboratory of Microbial Culture Collection and Application, Guangdong Open Laboratory of Applied Microbiology, Guangdong Institute of Microbiology, Guangzhou, China

**Keywords:** *Staphylococcus aureus*, ready-to-eat food, MLST, toxin genes, SEs

## Abstract

The aim of this study was to characterize the subtypes and virulence profiles of 69 *Staphylococcus aureus* isolates obtained from retail ready-to-eat food in China. The isolates were analyzed using multilocus sequence typing (MLST) and polymerase chain reaction (PCR) analysis of important virulence factor genes, including the staphylococcal enterotoxin (SE) genes (*sea*, *seb*, *sec*, *sed*, *see*, *seg*, *seh*, *sei*, *sej*), the exfoliative toxin genes (*eta* and *etb*), the toxic shock syndrome toxin-1 gene (*tst*), and the Panton-Valentine leucocidin-encoding gene (*pvl*). The isolates encompassed 26 different sequence types (STs), including four new STs (ST3482, ST3484, ST3485, ST3504), clustered in three clonal complexes and 17 singletons. The most prevalent STs were ST1, ST6, and ST15, constituting 34.8% of all isolates. Most STs (15/26, 57.7%) detected have previously been associated with human infections. All 13 toxin genes examined were detected in the *S. aureus* isolates, with 84.1% of isolates containing toxin genes. The three most prevalent toxin genes were *seb* (36.2%), *sea* (33.3%), and *seg* (33.3%). The classical SE genes (*sea*–*see*), which contribute significantly to staphylococcal food poisoning (SFP), were detected in 72.5% of the *S. aureus* isolates. In addition, *pvl*, *eta*, *etb*, and *tst* were found in 11.6, 10.1, 10.1, and 7.2% of the *S. aureus* isolates, respectively. Strains ST6 carrying *sea* and ST1 harboring *sec-seh* enterotoxin profile, which are the two most common clones associated with SFP, were also frequently detected in the food samples in this study. This study indicates that these *S. aureus* isolates present in Chinese ready-to-eat food represents a potential public health risk. These data are valuable for epidemiological studies, risk management, and public health strategies.

## Introduction

The facultative anaerobe *Staphylococcus aureus* is the leading cause of both nosocomial infections and community-acquired infections worldwide, causing many serious illnesses (Rodríguez-Lázaro et al., 2015; Yang et al., 2016). In addition, high rates of human nasal carriage, a high incidence of airborne spread, and the ability to survive for long periods on fomites contribute to making *S. aureus* an effective foodborne pathogen. Of particular concern is the fact that *S. aureus* is increasingly showing resistance to multiple antimicrobial agents. Methicillin-resistant *S. aureus* (MRSA) strains, including healthcare-acquired MRSA (HA-MRSA), community-acquired MRSA (CA-MRSA), and livestock-associated MRSA (LA-MRSA) strains, exhibit resistance to all β-lactam antibiotics through acquisition of the mobile staphylococcal cassette chromosome *mec* (SCC*mec*) and constitute a major health concern.

*Staphylococcus aureus* possesses various virulence factors that are implicated in pathogenesis, such as the staphylococcal enterotoxins (SEs), toxic shock syndrome toxin-1 (TSST-1), the exfoliative toxins A and B (ETA and ETB), and Panton-Valentine leukocidin (PVL) (Wang et al., 2012, 2014; Song et al., 2015). Among these, SEs and TSST-1 belong to a family of staphylococcal superantigens that cause the abnormal activation of T-cells (Yamamoto et al., 2013). The heat-stable SEs are associated with staphylococcal food poisoning (SFP) and other severe diseases (Yan et al., 2012). To date, at least 24 SEs (SEA–E, SEG–V, and SEX) have been identified (Yamamoto et al., 2013). Of these, types SEA–SEE are referred to as the classical SE types, and are reported to cause about 95% of SFP cases (Wang et al., 2012). The TSST-1 causes toxic shock syndrome (TSS) and neonatal TSS-like exanthematous disease (He et al., 2013; Yamamoto et al., 2013). Furthermore, ETA and ETB are epidermolytic proteases that digest the desmoglein 1 component of desmosomes, which disrupt stratum granulosum and subsequently cause blister formation and exfoliation of the epidermis (Yamamoto et al., 2013), a condition known as staphylococcal scalded skin syndrome (Song et al., 2015). Finally, PVL causes necrotizing pneumonia, sepsis, and severe tissue damage (Lina et al., 1999; Yamamoto et al., 2013). The presence of these virulence factors in *S. aureus* isolates from food may therefore pose a threat to public health.

Multilocus sequence typing (MLST) has been widely used to investigate the epidemiology of microbial populations (Xu et al., 2015; Wu et al., 2016). The method is based on the sequencing of a number of different housekeeping genes, with the resulting data made publicly available online. This allows the comparison of different isolates and their sequence types (STs) between laboratories, thus producing a powerful resource for global epidemiology (Maiden et al., 1998). Owing to these advantages, MLST has become an important and reliable technique for epidemiological analysis of *S. aureus*. For HA-MRSA, CA-MRSA, and LA-MRSA strains, they produce different STs, which help to identify them. The New York/Japan (ST5/SCC*mec* II) and Brazilian/Hungarian (ST239/SCC*mec* III) clones are pandemic HA-MRSA lineages (Yamamoto et al., 2013), while the Taiwanese (ST59/SCC*mec* IV or V), USA300 (ST8/SCC*mec* IV), European (ST80/SCC*mec* IV), and USA400 (ST1) clones are always associated with community-acquired infections (Yamamoto et al., 2013).

Ready-to-eat (RTE) food, such as cooked meat and poultry, cold vegetable dishes, cold noodles, and fried rice, are becoming more popular in China and their consumption has increased significantly. In our previous work, 69 *S. aureus* isolates were identified from Chinese RTE food samples, and the clonal lineages of the MRSA isolates were determined, including CA-MRSA [ST59/SCC*mec* IVa (*n* = 2), ST338/SCC*mec* V, ST1/SCC*mec* V], and LA-MRSA (ST9) (Yang et al., 2016). To assess the potential virulence and risk posed by these 69 *S. aureus* isolates from RTE food, we provide a phylogenetic framework for these isolates in the current study, based on MLST analysis and screened for the presence of virulence-associated genes.

## Materials and Methods

### *Staphylococcus aureus* Strains

We analyzed a total of 69 *S. aureus* isolates from 550 RTE food samples (cooked pork, cooked chicken, cooked duck, cold vegetable dishes in sauce, cold noodles, and fried rice/sushi) collected from markets in 24 Chinese cities from December 2011 to May 2014 (Yang et al., 2016). Of the 69 *S. aureus* isolates, seven were identified as methicillin-resistant, of which six were shown to be *mecA*-positive (Yang et al., 2016). All isolates were stored at -40°C until further analysis.

### Multilocus Sequence Typing

Of the 69 *S. aureus* isolates, the STs of the six *mecA*-positive MRSA isolates were determined in our previous work (Yang et al., 2016). In this study, the remaining 63 *S. aureus* isolates [62 methicillin-susceptible *S. aureus* (MSSA) and 1 *mecA*-negative MRSA] were characterized by MLST analysis. Chromosomal DNA was prepared using a Genomic DNA Extraction kit (Dongsheng Biotech, Guangzhou, China) following the manufacturer’s protocol, and MLST was carried out using previously reported primers that are specific for seven housekeeping genes: *arcC*, *aroE*, *glpF*, *gmk*, *pta*, *tpi*, and *yqiL*. The ST of each isolate was assigned according to the MLST database^1^. The associated clonal complex (CC) was calculated using the eBURST algorithm, which is used to cluster related STs^2^. A phylogenetic tree was generated using the unweighted pair-group method with arithmetic mean from the sequence data using MEGA 6.06.

### Detection of Virulence-Associated Genes

Thirteen toxin genes with reported contributions to virulence were selected, including nine major SE genes (*sea*, *seb*, *sec*, *sed*, *see*, *seg*, *seh*, *sei*, *sej*), the TSST-1 toxin gene (*tst*), the exfoliative toxin genes (*eta* and *etb*), and the PVL-encoding gene (*pvl*). All examined 69 *S. aureus* isolates were screened for the presence of 13 toxin genes using previously described primers (Lina et al., 1999; Noguchi et al., 2006; Peles et al., 2007; Wang et al., 2012; Supplementary Table S1). The 25 μL reaction mixtures contained 12.5 μL of 2× GoldStar *Taq* MasterMix (Cwbio, Beijing, China), 0.5 μL of each primer (10 μM), 4 μL of extracted genomic DNA, and sterile deionized water. Polymerase chain reaction (PCR) was performed using a T-Professional thermocycler (Biometra, Gottingen, Germany) under the following conditions: initial denaturation at 95°C for 5 min, followed by 35 cycles of 95°C for 45 s, 58°C for 45 s, and 72°C for 60 s, and a final extension step at 72°C for 10 min. A 6 μL aliquot of each amplicon was resolved on a 1.5% agarose gel (Invitrogen, Paisley, United Kingdom) with GoldView Nucleic Acid Stain (SBS Genetech Co., Ltd., Beijing, China). Gels were examined using the ImageQuant 350 system (GE Healthcare, Chalfont St. Giles, United Kingdom).

## Results

### Multilocus Sequence Typing of *Staphylococcus aureus* Isolates

**FIGURE 1:**
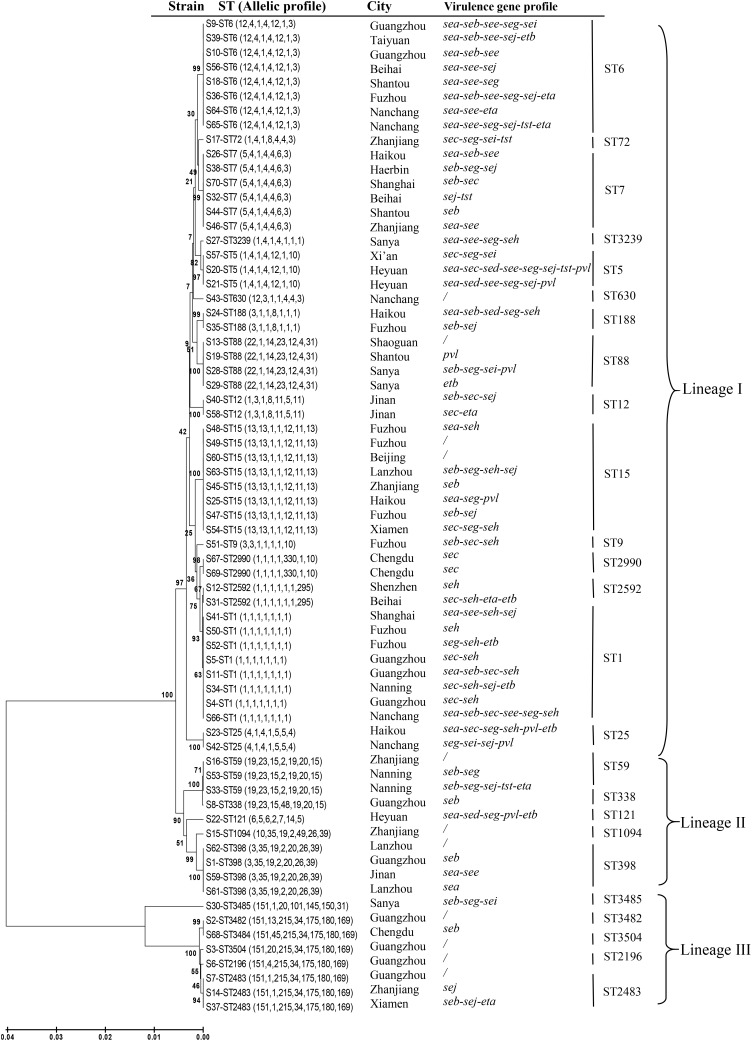
Unweighted pair-group method with arithmetic mean tree of the seven gene fragments examined during multi-locus sequence typing of *Staphylococcus aureus* isolates from ready-to-eat food in China.

In addition to the previously determined STs of the six *mecA*-positive MRSA isolates, a total of 26 distinct STs were identified, including four novel STs (ST3482, ST3484, ST3485, and ST3504) (**Table 1**). The number of alleles for the seven housekeeping genes ranged from 1 to 330. The most commonly detected allelic profiles were ST1, ST6, and ST15 (8/69, 11.6% for each). With the exception of ST9, ST72, ST121, ST338, ST630, ST1094, ST2196, ST3239, and the four novel STs, the remainder of the STs included more than one isolate. Of these, ST1, ST59, and ST25 included both MRSA and MSSA isolates. Based on eBURST analysis, three CCs were identified, including CC1 (ST1, ST2592), CC2483 (ST2483, ST2196, ST3482, ST3484, ST3504), and CC59 (ST59, ST338). The two predominant CCs (CC1 and CC2483) included 14.5% (9/62) and 11.3% (7/62) of the MSSA isolates, respectively, while CC59 accounted for the majority of MRSA isolates (3/7, 42.9%). A phylogenetic tree based on the seven concatenated sequences generated during MLST showed clustering among the 69 RTE food isolates and indicated the relatedness between the STs (**Figure 1**).

**Table 1:** Allelic profiles of *Staphylococcus aureus* isolates from ready-to-eat food as determined by multilocus sequence typing.

Clonal complex	MSSA	MRSA	MLST (no. of isolates)	arc	aro	glp	gmk	pta	Tpi	yqi
CC1 (10)	7	1^a^	ST1 (8)	1	1	1	1	1	1	1
	2		ST2592 (2)	1	1	1	1	1	1	295
CC2483 (7)	3		ST2483 (3)	151	1	215	34	175	180	169
	1		ST2196 (1)	151	4	215	34	175	180	169
	1		ST3482 (1)^∗^	151	13^∗^	215	34	175	180	169
	1		ST3484 (1)^∗^	151	45^∗^	215	34	175	180	169
	1		ST3504 (1)^∗^	151	20^∗^	215	34	175	180	169
CC59 (4)	1	2^a^	ST59 (3)	19	23	15	2	19	20	15
		1^a^	ST338 (1)	19	23	15	48	19	20	15
Singletons	8		ST6 (8)	12	4	1	4	12	1	3
	8		ST15 (8)	13	13	1	1	12	11	13
	6		ST7 (6)	5	4	1	4	4	6	3
	4		ST88 (4)	22	1	14	23	12	4	31
	4		ST398 (4)	3	35	19	2	20	26	39
	3		ST5 (3)	1	4	1	4	12	1	10
	2		ST12 (2)	1	3	1	8	11	5	11
	1	1	ST25 (2)	4	1	4	1	5	5	4
	2		ST188 (2)	3	1	1	8	1	1	1
	2		ST2990 (2)	1	1	1	1	330	1	10
		1^a^	ST9 (1)	3	3	1	1	1	1	10
	1		ST72 (1)	1	4	1	8	4	4	3
	1		ST121 (1)	6	5	6	2	7	14	5
	1		ST630 (1)	12	3	1	1	4	4	3
	1		ST1094 (1)	10	35	19	2	49	26	39
		1^a^	ST3239 (1)	1	4	1	4	1	1	1
	1		ST3485 (1)^∗^	151	1	20	101	145	150	31^∗^

*^∗^Novel alleles and novel sequence type.*^a^*The allelic profiles of the six mecA-positive methicillin-resistant S. aureus isolates were reported previously by Yang et al. (2016)*.

### Distribution of Toxin Genes

All 13 toxin genes examined were detected in the 69 *S. aureus* isolates. In total, toxin genes were identified in 84.1% (58/69) of *S. aureus* isolates, and 49.3% (34/69) of isolates contained more than three toxin genes. As shown in **Table 2**, 81.2% of *S. aureus* isolates harbored SE genes and the three most prevalent were *seb* (36.2%, 25/69), *sea* (33.3%, 23/69), and *seg* (33.3%, 23/69). In addition, *pvl*, *eta*, *etb*, and *tst* were found in 11.6, 10.1, 10.1, and 7.2% of the *S. aureus* isolates, respectively.

**Table 2:** Distribution of 13 toxin genes in *Staphylococcus aureus* isolates from retail ready-to-eat food in China.

Type of products	*sea*	*seb*	*sec*	*sed*	*see*	*seg*	*seh*	*sei*	*sej*	*tst*	*eta*	*etb*	*pvl*	>1	>3	SE *gene positive*	*sea*–*see*
MSSA (*n* = 62)	20	20	14	4	14	18	13	6	17	4	6	6	7	51 (82.3)	29 (46.8)	49 (79.0)	43 (69.4)
MRSA (*n* = 7)	3	5	3	0	2	5	4	0	1	1	1	1	1	7 (100.0)	5 (71.4)	7 (100.0)	7 (100.0)
Total	23	25	17	4	16	23	17	6	18	5	7	7	8	58 (84.1)	34 (49.3)	56 (81.2)	50 (72.5)

Overall, the 69 *S. aureus* isolates displayed 59 different toxin gene profiles, with one to eight toxin genes found in each isolate in different combinations. The most prevalent toxin gene profile was the single enterotoxin gene *seb* (7.2%, 5/69). The *sea*-*see* combination, with or without additional toxin genes, occurred in 16 isolates, and it was the most common combination observed. As for the distribution of toxin genes among the *S. aureus* isolates based on the food categories, the SE genes, except *sed* and *sei*, were detected in isolates from all six food categories. All *S. aureus* isolates contained the classic SE genes in samples collected from cold vegetable dishes in sauce and fried rice/sushi, and these two kinds of food, along with cooked chicken, were also the major sources of isolates containing multiple toxin genes (**Table 3**).

**Table 3:** Distribution of 13 toxin genes in *Staphylococcus aureus* isolates arranged by different food categories.

Type of products	*sea*	*seb*	*sec*	*sed*	*see*	*seg*	*seh*	*sei*	*sej*	*tst*	*eta*	*etb*	*pvl*	>1	>3	SE *gene positive*	*sea*–*see positive*
Cooked pork(*n* = 15)	4	6	3	0	2	1	1	1	3	0	0	1	0	10 (66.7)	5 (33.3)	9 (60.0)	9 (60.0)
Cooked chicken (*n* = 15)	4	8	4	2	3	7	4	2	5	0	0	3	2	14 (93.3)	10 (66.7)	14 (93.3)	12 (80.0)
Cooked duck (*n* = 17)	4	4	3	1	3	5	3	2	5	3	3	1	3	13 (76.5)	5 (29.4)	13 (76.5)	10 (58.8)
Cold vegetable dishes in sauce(*n* = 6)	4	1	2	0	3	3	1	0	1	1	2	1	1	6 (100.0)	4 (66.7)	6 (100.0)	6 (100.0)
Cold noodles (*n* = 10)	5	5	2	1	4	5	5	0	2	0	1	1	1	9 (90.0)	6 (60.0)	8 (80.0)	7 (70.0)
Fired rice/sushi (*n* = 6)	2	1	3	0	1	2	3	1	2	1	1	0	1	6 (100.0)	4 (66.7)	6 (100.0)	6 (100.0)
Total	23	25	17	4	16	23	17	6	18	5	7	7	8	58 (84.1)	34 (49.3)	56 (81.2)	50 (72.5)

### Association Between STs and Toxin Genes

Some associations were noted between certain STs and toxin genes. For example, each isolate belonging to CC1 contained s*eh*, while the *sec*-*seh* combination, with or without additional toxin genes, occurred in 50% of the CC1 isolates. The combination of *sea*-*see* with additional toxin genes was found in all eight isolates assigned to ST6. Furthermore, half of the ST6 isolates also harbored *eta* or *etb*. Eight isolates contained *pvl* and mainly belonged to three STs (ST5, ST25, and ST88). In addition, all of the isolates belonging to ST5, ST6, or ST25 carried more than three toxin genes.

## Discussion

Human infections caused by foodborne *S. aureus* strains have been previously reported (Jones et al., 2002). Therefore, the potential role of food in the dissemination of successful *S. aureus* lineages cannot be ignored. Our previous study reported the existence of two lineages of MRSA in these RTE food: CA-MRSA [ST59/SCC*mec* IVa (*n* = 2), ST338/SCC*mec* V, ST1/SCC*mec* V], and LA-MRSA (ST9) (Yang et al., 2016). Both ST59 and ST338 belong to CC59, which is the prevalent CA-MRSA clone in China and other Asian countries (Li et al., 2013; Chen and Huang, 2014). Along with three CC59 MRSA isolates, we also identified a single ST59 MSSA isolate among the RTE samples. In China, ST59 and ST338 are the first and second most dominant STs in cases of pediatric community-acquired pneumonia (Geng et al., 2010).

The strain ST398 was the first detected and most widespread LA-MRSA clone in Europe and North America (Petinaki and Spiliopoulou, 2012), in contrast, ST9 is the most prevalent LA-MRSA clone in most Asian countries (Cui et al., 2009; Wagenaar et al., 2009; Petinaki and Spiliopoulou, 2012) and ST398 MSSA is predominant in Asian countries, including China (Asai et al., 2012; Li et al., 2015). This was confirmed in our study. In addition to the previously identified ST9 LA-MRSA isolate, we identified four ST398 MSSA isolates, but no ST398 MRSA isolates were detected. The identification of ST398 MSSA isolates is of particular concern because human infections caused by MSSA ST398 strains have recently been described (David et al., 2013; Li et al., 2015).

The strain ST1 was the most common *S. aureus* clone causing SFP in Korea (Cha et al., 2006). However, in China, the ST6 strain carrying *sea* was the most dominant clone among isolates associated with SFP outbreaks and the ST1 strain with *sec*-*seh* enterotoxin profile was also frequently detected (Yan et al., 2012). In the present study, ST6 was found to be one of the most common STs. Furthermore, each isolate belonging to ST6 contained *sea*, and half of them also harbored *eta* and *etb*. In addition to the ST1 MRSA isolate, seven ST1 MSSA isolates were also identified in this study. These eight ST1 isolates, together with the two ST2592 MSSA isolates, constituted the predominant CC1. Moreover, half of the CC1 isolates exhibited the *sec*-*seh* enterotoxin profile with or without additional toxin genes. These results suggest that food is a potential source of *S. aureus* strains, which is significantly relevant on a clinical basis. With the exception of CC2483, ST9, ST12, ST1094, ST2592, ST2990, ST3239, and ST3485, all of the STs or CCs identified in this study have also been linked to bacteremia in China (He et al., 2013). Thus, these types of *S. aureus* isolates found in Chinese RTE food in the current study may represent a risk to human health.

Toxins are generally regarded as one of the main factors in the virulence of *S. aureus* worldwide, and for this reason, it is important to determine their prevalence in isolates from food with respect to assessing public health risks. In the current study, the prevalence of *S. aureus* isolates containing SE genes was higher than in several previous studies from food products in both China and other countries (Hammad et al., 2012; Vázquez-Sánchez et al., 2012; Wang et al., 2012, 2014). The classical SE genes (*sea*–*see*), which contribute significantly to SFP, were detected in 72.5% of the *S. aureus* isolates.

As for the single SE gene, *seb*, *sea*, and *seg* were the most frequently detected, followed by *sej*, *seh*, and *sec* in this study. Notably, SEB has been listed as a biological warfare agent because it can be toxic at very low concentrations (Varshney et al., 2009; Yamamoto et al., 2013). Recent studies indicated that SEB showed lethality in a rabbit model of pneumonia, and may suppress the motility of polymorphonuclear neutrophils, allowing MRSA to invade and damage tissues (Yamamoto et al., 2013). Infection by *S. aureus* strains that produce SEB may lead to serious diseases (Varshney et al., 2009). It is a concern that *seb* was prevalent in both the MSSA and MRSA isolates in the present study; likewise, *seb* was predominantly distributed in *S. aureus* isolates in the previous studies from food products in China and Vietnam (Huong et al., 2010; Wang et al., 2014).

Furthermore, SEA has been considered the most common cause of SFP worldwide (Pinchuk et al., 2010; Vázquez-Sánchez et al., 2012). The dominance of *sea* observed in the present study is consistent with previous findings (Vázquez-Sánchez et al., 2012; Zhang et al., 2013) and provides further evidence that this gene is widely distributed among *S. aureus* strains from food. In China, *sea* was reported to be the most commonly detected toxin gene in clinical *S. aureus* isolates associated with food poisoning outbreaks (Yan et al., 2012). In addition, the high prevalence of *seg* observed in this study was similar to the previous reports in China (Wang et al., 2012, 2014). However, *seh* and *sej*, which were also commonly identified in the present study, were not detected in the studies of Wang et al. (2012, 2014).

In this study, 11.6% of *S. aureus* isolates carried *pvl*. Among them, only one MRSA isolate, which was *mecA*-negative, contained *pvl*. As for *pvl* carriage by MRSA isolates, the previously reported rate varied greatly. While an absence of *pvl* in MRSA isolates has been reported (Fessler et al., 2011; Vestergaard et al., 2012), other studies show a high *pvl* carriage rate among MRSA isolates from food in China (Wang et al., 2014) and other countries (Pu et al., 2009; Hanson et al., 2011). Because *pvl*-positive *S. aureus* strains can cause necrotizing pneumonia, bloodstream infection, and soft-tissue pyogenic infection (Hammad et al., 2012), their potential to cause infections in humans through the food chain needs attention.

A novel finding in this study is the observed high rate of *S. aureus* strains carrying *eta*, *etb*, and *tst.* Previous studies in China have never reported the identification of *etb* and *tst* in *S. aureus* isolates from food, and the detected *eta* level was very low (Wang et al., 2012, 2014; Song et al., 2015). It has been reported that *S. aureus* clones containing *eta*, *etb*, and *tst* are increasingly responsible for severe infections (Xie et al., 2011; He et al., 2013; Yamamoto et al., 2013; Song et al., 2015). Thus, the presence of these isolates in RTE food indicates a potential risk to public health.

Nowadays, it is well established that the production of SEs in food is affected by several factors and the formation of SEs in food environments is significantly different from that in cultures of pure *S. aureus* (Schelin et al., 2011). In our study, we only used PCR analysis to determine the presence of toxin genes in pure *S. aureus* strains. These data are valuable and can represent virulence potential of *S. aureus* isolates to some extent.

Additionally, it was reported that food poisoning by *S. aureus* toxins does not occur until the level of the pathogen reaches 100,000 to 1,000,000/g (Food and Drug Administration [FDA], 2001; Hammad et al., 2012). The *S. aureus* contamination levels of the positive food samples had been determined in our previous work, these were mostly in the range of 0.3–10 most probable number (MPN)/g, with five samples exceeding 10 MPN/g (Yang et al., 2016). However, the RTE foods of this study are consumed without further treatment, the microbial load will increase over time, which represents a potential health hazard to humans.

## Conclusion

In summary, to the best of our knowledge, this is the first comprehensive study of the subtypes and presence of virulence-associated genes in *Staphylococcus aureus* isolates from retail ready-to-eat (RTE) food in most regions in China. Our findings revealed a high prevalence of *S. aureus* isolates containing toxin genes in RTE food in China, and most of the sequence types detected in this study have previously been associated with human infections. The three most common toxin genes were *sea*, *seb*, and *seg*, and other important toxin genes, including *pvl*, *eta*, *etb*, and *tst*, were also detected in these *S. aureus* isolates. Overall, our study indicated that these *S. aureus* isolates present in Chinese RTE food represents a potential public health risk.

## Author Contributions

Conceived and designed the experiments: XY, QW, and JZ. Performed the experiments: XY and SY. Analyzed the data: XY, SY, SW, and DR.

## Conflict of Interest Statement

The authors declare that the research was conducted in the absence of any commercial or financial relationships that could be construed as a potential conflict of interest.

## Footnotes

1 http://www.mlst.net/

2 http://saureus.mlst.net/eBURST
