# Supplementary material for: Multilocus Sequence Typing and Virulence-Associated Gene Profile Analysis of Staphylococcus aureus Isolates From Retail Ready-to-Eat Food in China
Source: Front Microbiol. 2018 Mar 13;9:197. doi: 10.3389/fmicb.2018.00197 (PMC5890145; doi:10.3389/fmicb.2018.00197)
Supplement: Supplementary file 1 [file Table_1.DOCX]

**Supporting Information**

**Table S1.** Sequences of primers used for detection of toxin genes

| Gene target | Primer sequences (5’-3’) | References |
| --- | --- | --- |
| 1-*sea* | F: GGT TAT CAA TGT GCG GGT GG | Peles et al. (2007); Wang et al. (2012). |
|  | R: CGG CAC TTT TTT CTC TTC GG |  |
| 2-*seb* | F: GTA TGG TGG TGT AAC TGA GC |  |
|  | R: CCA AAT AGT GAC GAG TTA GG |  |
| 3-*sec* | F: AGA TGA AGT AGT TGA TGT GTA TGG |  |
|  | R: CAC ACT TTT AGA ATC AAC CG |  |
| 4-*sed* | F: CCA ATA ATA GGA GAA AAT AAA AG |  |
|  | R: ATT GGT ATT TTT TTT CGT TC |  |
| 5-*see* | F: AGG TTT TTT CAC AGG TCA TCC |  |
|  | R: CTT TTT TTT CTT CGG TCA ATC |  |
| 6-*seg* | F: TGC TAT CGA CAC ACT ACA ACC |  |
|  | R: CCA GAT TCA AAT GCA GAA CC |  |
| 7-*seh* | F: CGA AAG CAG AAG ATT TAC ACG |  |
|  | R: GAC CTT TAC TTA TTT CGC TGT C |  |
| 8-*sei* | F: GAC AAC AAA ACT GTC GAA ACT G |  |
|  | R: CCA TAT TCT TTG CCT TTA CCA G |  |
| 9-*sej* | F: CAT CAG AAC TGT TGT TCC GCT AG |  |
|  | R: CTG AAT TTT ACC ATC AAA GGT AC |  |
| 10-*tst* | F: ACC CCT GTT CCC TTA TCA TC |  |
|  | R: TTT TCA GTA TTT GTA ACG CC |  |
| 11-*eta* | F: ATA TCA ACG TGA GGG CTC TAG TAC | Noguchi et al. (2006); Wang et al. (2012). |
|  | R: ATG CAG TCA GCT TCT TAC TGC TA |  |
| 12-*etb* | F: CAC ACA TTA CGG ATA ATG CAA G |  |
|  | R: TCA ACC GAA TAG AGT GAA CTT ATC T |  |
| 13-*pvl* | F: ATCATTAGGTAAAATGTCTGGACATGATCCA | Lina et al. (1999). |
|  | R: GCATCAASTGTATTGGATAGCAAAAGC |  |
